# Dietary habits and the presence and degree of asymptomatic diverticular disease by magnetic resonance imaging in a Western population: a population-based cohort study

**DOI:** 10.1186/s12986-021-00599-4

**Published:** 2021-07-16

**Authors:** Esther Askani, Susanne Rospleszcz, Theresa Rothenbacher, Nina Wawro, Helmut Messmann, Carlo N. De Cecco, Ricarda von Krüchten, Charlotte Kulka, Lena S. Kiefer, Wolfgang Rathmann, Annette Peters, Christopher L. Schlett, Fabian Bamberg, Jakob Linseisen, Corinna Storz

**Affiliations:** 1 Department of Diagnostic and Interventional Radiology, Medical Center – University of Freiburg, Freiburg, Germany; 2 Chair of Epidemiology, Institute for Medical Information Processing, Biometry, and Epidemiology, Ludwig-Maximilians-University Munich, Munich, Germany; 3 Institute of Epidemiology, Helmholtz Center Munich, German Research Center for Environmental Health, Neuherberg, Germany; 4 Department of Diagnostic and Interventional Radiology, University Hospital Tuebingen, Tuebingen, Germany; 5 Independent Research Group Clinical Epidemiology, Helmholtz Center Munich, German Research Center for Environmental Health, Neuherberg, Germany; 6 Chair of Epidemiology, Ludwig-Maximilians University of Munich, UNIKA-T Augsburg, Augsburg, Germany; 7 Department of Internal Medicine III, Klinikum Augsburg, Augsburg, Germany; 8 Division of Cardiothoracic Imaging, Department of Radiology and Imaging Sciences, Emory University Hospital, Atlanta, GA USA; 9 Institute of Biometrics and Epidemiology, German Diabetes Center, Duesseldorf, Germany; 10 German Center for Cardiovascular Disease Research (DZHK E.V.), Munich, Germany; 11 Department of Neuroradiology, Medical Center – University of Freiburg, Faculty of Medicine, University of Freiburg, Breisacher Str. 64, 79106 Freiburg, Germany

**Keywords:** Diverticular disease, MRI, Dietary habits

## Abstract

**Background:**

Despite the worldwide burden of diverticular disease, the connections between diverticular disease and dietary habits remain poorly understood, particularly in an asymptomatic representative sample. We investigated the association between asymptomatic diverticular disease as assessed by magnetic resonance imaging (MRI) and dietary habits in a Western study cohort.

**Methods:**

Participants from a cross-sectional sample of a population-based cohort study underwent whole-body 3T-MRI including an isotropic VIBE-Dixon sequence. The presence and extent of diverticular disease was assessed in blinded fashion. Habitual dietary intake was recorded using a blended approach, applying 24-h food lists and a food-frequency questionnaire. Traditional cardiometabolic risk factors were obtained by interviews and medical examination. Univariate and multivariate associations were calculated.

**Results:**

A total of 308 subjects were included in this analysis (56% male, 56.4 ± 9.1 years). 39.9% had any form of diverticular disease and 15.3% had advanced asymptomatic diverticular disease. After adjustment for age, sex and total energy intake a higher intake of fiber and vegetables was associated with a lower odds for asymptomatic diverticular disease (fiber: OR 0.68 95% CI [0.48, 0.95]; vegetables: OR 0.72 95% CI [0.53, 0.97]) and an increased intake of meat was associated with an approximately two-fold higher odds for advanced asymptomatic diverticular disease (OR 1.84 95% CI [1.13, 2.99]). However, after additional adjustment for body-mass-index (BMI), alcohol consumption, smoking behavior and physical activity only a high fiber and vegetables intake remained significantly associated with lower odds of asymptomatic diverticular disease.

**Conclusion:**

Our results indicate that a high-fiber diet and increased intake of vegetables is associated with lower odds of having asymptomatic diverticular disease, independent of age, sex, total energy intake, BMI and other life-style factors.

**Supplementary Information:**

The online version contains supplementary material available at 10.1186/s12986-021-00599-4.

## Background

Diverticular disease is a common condition in Western countries with increasing prevalence [1–4]. Asymptomatic diverticular disease refers to the mere presence of diverticula, which are defined as small protrusions of colonic mucosa through the outer muscular layers at sites of vascular perforation [5]. Complications of diverticular disease resulting in clinically significant diverticulosis include diverticulitis, diverticular bleeding, perforation or obstruction [6–9]. Approximately 4–40% of patients with diverticular disease develop acute or chronic complications [10]. The estimated mortality rate due to diverticular disease and its complications amounts to 2.5 per 100,000 per year [11]. Furthermore diverticular disease is the fifth most important gastrointestinal disease in terms of healthcare costs in the United States [11]. Today, in industrialized western countries, approximately 5% of people in their fifth decade and 50% of those in their ninth decade are affected with asymptomatic diverticular disease [3], indicating that the overall prevalence increases with age but also that its medical and economic relevance is likely to increase further as the population ages [11, 12].

Although little is known about the etiopathogenesis of diverticular disease [13], it has been observed that western and industrialized countries like the United States, Europe and Australia have a considerably higher prevalence of diverticular disease compared to countries such as Africa and Asia, which have prevalence rates of less than 0.5% [4]. This fact supports the consideration, that life-style factors may be responsible for the development of diverticular disease. Some risk factors have been identified unanimously by different studies to promote the development of diverticular disease whereas others are less understood and are still controversially discussed. Age, body mass index (BMI), waist circumference and waist-to-hip-ratio have been identified as risk factors for diverticular disease [14–18]. While there is evidence that the risk of diverticular disease was lower among vegetarians or vegans compared to meat eaters [19, 20], it was also observed that a high-fiber diet was associated with a higher prevalence of diverticula [16], whereas nut, corn and popcorn consumption did not increase the risk of diverticulosis or diverticular complications [21]. As such, despite the burden of diverticular disease the connections between diverticular disease and dietary habits remain poorly understood, particularly in an asymptomatic representative sample.

Previously we showed that magnetic resonance imaging (MRI) represents a valid, reproducible, non-invasive modality for the assessment of asymptomatic diverticular disease and our initial results confirmed that diverticular disease is common in a Western general population [22]. The objective of the present study was to determine the association of habitual dietary intake and the presence of asymptomatic diverticular disease as characterized by MRI in a sample of a general Western population. Our hypothesis was that a high-fiber diet and an increased intake of vegetables is associated with a lower degree of asymptomatic diverticular disease, whereas an increased meat intake is linked with a higher degree of asymptomatic diverticular disease.

## Methods

### Study design and recruitment of participants

This study was designed as a cross-sectional study, embedded in a population-based cohort from the ‘Cooperative Health Research in the Augsburg Region, Germany’ (KORA), as described elsewhere [23, 24].

KORA is a population-based research platform with subsequent follow-up studies in the fields of epidemiology, health economics, and health research. Within KORA, starting from 1996 four cross-sectional surveys S1 to S4 have been performed at five year interval, each comprising of an independent random sample, and serving as cohorts for long-term follow-up studies and as a pool for nested case–control and case-cohort studies [24].

The study population of the present study was recruited from the second follow-up of the KORA S4 cohort (FF4), which took place between June 2013 and September 2014 and comprises N = 400 participants from the FF4 study who were enrolled in an MRI substudy [23].

In brief, participants without prior cardiovascular disease underwent a whole-body MRI examination, if no contraindications to MRI and administration of gadolinium contrast were present. Participants with the following conditions were excluded from the study: > 72 years of age, participants with prior cardiovascular diseases (e.g. stroke, myocardial infarction or revascularization), non-MRI-suitable implanted devices (e.g. cardiac pacemaker or implantable defibrillator, cerebral aneurysm clip, neural stimulator, any type of ear implant, ocular foreign body), pregnant or breast feeding participants, or participants with claustrophobia, known allergy against gadolinium compounds, or impaired renal function (serum creatinine ≥ 1,3 mg/dl). All subjects underwent MRI within 3 months after their FF4 clinical examination [23].

### Nutrition and dietary habits

Within the FF4 follow-up cohort of the KORA S4 health survey data on nutrition and dietary habits were collected until before the end of November 2014 [25]. Usual dietary intake was estimated with a blended approach combining repeated 24-h-food lists and a food frequency questionnaire (FFQ) [25]. Information about food consumption of the participants from up to three 24-h food lists [26] and a FFQ [27] were available to estimate consumption probabilities. We followed a two-step procedure, where the consumption probability and the amount of consumption on consumption days were estimated separately [25, 28]. Both models included the same covariates, thereby linking the two parts. Consumption amounts on a consumption day were estimated based on the detailed food intake data collected in the Bavarian Food Consumption survey II [29]. The usual intake was then derived as the product of the probability of consuming a certain food and the usual amount consumed on a consumption day. Food intake data were aggregated to food groups and subgroups. Energy and nutrient intake data were calculated by linking the usual intake data to the German food composition database BLS, Version 3.02.

### Other covariates

During the FF4 follow-up information on sociodemographic variables and lifestyle factors was collected in an extensive standardized face-to-face interview. Furthermore, all participants underwent anthropometric measurements [24].

BMI was defined as the value from the mass of the participant in kilogram divided by its height squares in meters and participants were categorized into (a) normal (BMI < 25 kg/m^2^), (b) overweight (BMI 25 to < 30 kg/m^2^) and (c) obese (BMI ≥ 30 kg/m^2^), according to the definition of the world health organization [30].

Smoking behavior was defined as never-, ex- and current smoker (current regular or sporadic cigarette smoking) [31]. Alcohol consumption was assessed in g/d [32]. Physical activity was categorized as physically active (regular physical activity ≥ 1 h/week) or physically inactive (irregular physical activity < 1 h/week, almost no physical activity and no physical activity at all) [31, 33].

### Magnetic resonance imaging protocol and imaging analysis

All participants underwent an identical imaging protocol on a 3 Tesla Magnetom Skyra (Siemens Healthineers, Erlangen, Germany), as detailed previously [23, 24].

### Assessment of diverticular disease

For the assessment of asymptomatic diverticular disease, a two-point T1 weighted isotropic VIBE-Dixon gradient-echo sequence of the abdomen was employed (TR 4.06 ms, TE 1.26, 2.49 ms, flip angle 9°, partition thickness 1.7 mm, isotropic in-plane resolution 1.7 mm), as described previously [22, 23].

Diverticular disease was assessed in every colonic segment. The colonic segments were divided into the caecum, ascending colon, transverse colon, descending colon and sigmoid colon and screened for the assessment of diverticula by two independent and blinded readers [22]. A graded-scale system was used to classify the extent of colonic diverticula according to the number of diverticula [34]: grade 1 = no diverticular disease, grade 2 = mild diverticular disease with at least 1 but < 6 diverticula in at least one colonic segment, grade 3 = advanced diverticular disease with ≥ 6 diverticula in at least one segment of the colon [22, 34].

### Statistical analysis

Demographics, risk factors, and nutrition data of the participants are presented as arithmetic mean and standard deviation for continuous covariates and as counts and percentages for categorical covariates. Overall differences in these covariates between grades of diverticular disease were evaluated by ANOVA or χ^2^-Test, where appropriate. Differences in these covariates between participants with advanced diverticular disease and no diverticular disease were determined by t-Test or χ^2^-Test. Associations of nutrition data to the presence of diverticular disease were assessed by logistic regression models adjusted for age, sex and total caloric energy intake in a Model 1 and additionally adjusted for BMI, physical activity, smoking behavior and alcohol consumption in a Model 2. As measures of association, odds ratios (OR) with corresponding 95%-confidence intervals (CI) were calculated per standard deviation of the variable of interest. All analyses were conducted with R v3.6.3 [35].

## Results

**Fig. 1:**
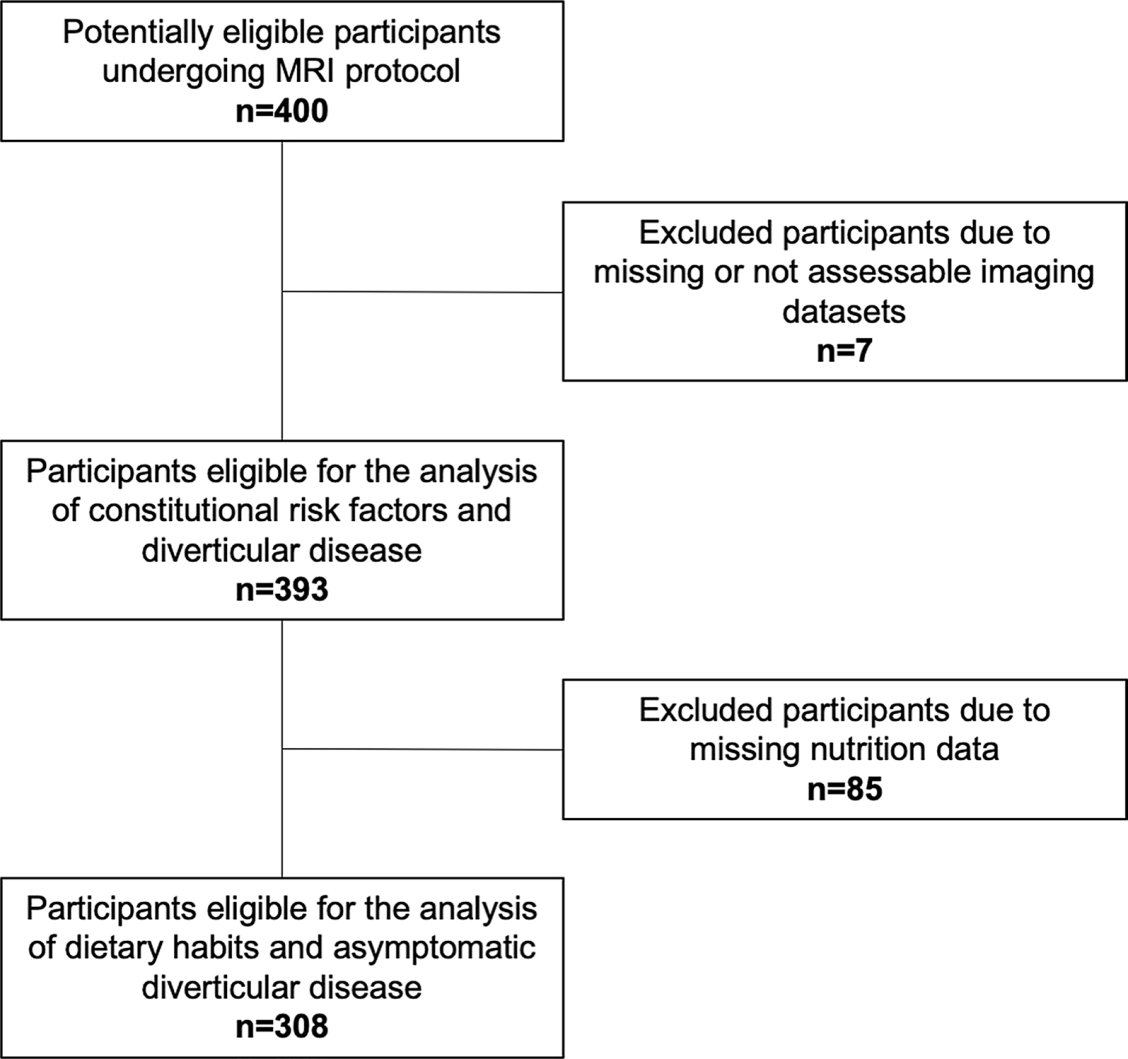
Study flowchart of participant inclusion and exclusion

Among 400 enrolled participants from the KORA study, 85 were excluded from the analysis due to missing information on dietary intake and 7 were excluded due to missing or not assessable imaging datasets (Fig. 1). In total, 308 participants (55.8% male, 56.4 ± 9.1 years) were eligible for the analysis of dietary habits and diverticular disease. Demographics of the study population are provided in Table 1. No difference was found between participants with and without available dietary intake data (Additional file 1: Table S1).

**Table 1:** Participants’ demographic and risk factors by grade of asymptomatic diverticular disease

	All	No diverticular disease	Mild diverticular disease	Advanced diverticular disease	*p* value (all)	*p* value advanced versus no diverticular disease
	N = 308	N = 185 (60.1%)	N = 76 (24.7%)	N = 47 (15.3%)		
Age (years)	56.4 ± 9.1	54.5 ± 8.7	57.0 ± 9.2	62.9 ± 7.2	** < 0.001**	** < 0.001**
Men	172 (55.8%)	102 (55.1%)	40 (52.6%)	30 (63.8%)	0.456	0.726
BMI (kg/m^2^)	28.0 ± 5.0	27.6 ± 5.0	27.4 ± 4.3	30.5 ± 5.3	**0.001**	**0.001**
Normal	89 (28.9%)	56 (30.3%)	25 (32.9%)	8 (17.0%)	**0.004**	**0.007**
Overweight	127 (41.2%)	78 (42.2%)	35 (46.1%)	14 (29.8%)		
Obese	92 (29.9%)	51 (27.6%)	16 (21.1%)	25 (53.2%)		
Alcohol (g/d)	18.2 ± 23.9	17.8 ± 24.9	18.5 ± 23.0	19.7 ± 21.2	0.879	0.623
Physical activity						
Inactive	122 (39.6%)	72 (38.9%)	32 (42.1%)	18 (38.3%)	0.874	1.000
Active	186 (60.4%)	113 (61.1%)	44 (57.9%)	29 (61.7%)		
Smoking						
Never-smoker	114 (37.0%)	72 (38.9%)	26 (34.2%)	16 (34.0%)	0.568	0.279
Ex-smoker	135 (43.8%)	76 (41.1%)	34 (44.7%)	25 (53.2%)		
Smoker	59 (19.2%)	37 (20.0%)	16 (21.1%)	6 (12.8%)		
Total cholesterol (mg/dl)	217.4 ± 36.3	212.9 ± 34.9	222.1 ± 39.1	227.7 ± 35.0	**0.019**	**0.025**
HDL cholesterol (mg/dl)	62.5 ± 17.8	63.0 ± 17.7	63.7 ± 19.2	58.2 ± 15.3	0.199	0.196
LDL cholesterol (mg(dl)	139.2 ± 33.5	133.9 ± 33.5	143.8 ± 33.3	152.6 ± 29.8	**0.001**	**0.001**
Triglycerides (mg/dl)	128.2 ± 79.7	126.9 ± 88.7	120.7 ± 67.4	145.2 ± 55.6	0.240	0.321
Vitamin D (ng/ml)	23.1 ± 11.4	22.2 ± 11.5	24.8 ± 11.3	24.2 ± 10.8	0.196	0.557
Systolic blood pressure (mmHg)	120.0 ± 16.4	117.6 ± 15.6	120.5 ± 17.6	128.9 ± 14.6	** < 0.001**	** < 0.001**
Diastolic blood pressure (mmHg)	74.8 ± 10.0	74.6 ± 9.3	73.8 ± 11.3	77.5 ± 10.0	0.111	0.143
Antihypertensive medication	84 (27.3%)	34 (18.4%)	24 (31.6%)	26 (55.3%)	** < 0.001**	** < 0.001**
Antithrombotic drugs	20 (6.5%)	4 (2.2%)	11 (14.5%)	5 (10.6%)	** < 0.001**	**0.037**

Bold values denote statistical significance at the *p* < 0.05 level

Variables as mean and standard deviation or number and percentage. *p* values from t-test or χ^2^-test, where appropriate. BMI = body mass index, HDL = high-density lipoprotein, LDL = low-density lipoprotein

### Prevalence of diverticular disease based on MRI

Overall, the prevalence of asymptomatic diverticular disease was high (prevalence: 123/308, 39.9%) with the highest proportion categorized as mild diverticular disease (24.7%) and 15.3% categorized as advanced diverticular disease.

### Association between habitual dietary intake and diverticular disease

The mean intake of selected food groups, energy and nutrients per day in participants without and with asymptomatic diverticular disease according to the grade is detailed in Table 2. The mean energy intake of all 308 participants with information about dietary habits was 1836.5 ± 409.3 kcal/day.

**Table 2:** Mean intake of selected food/nutrients per day in participants without and with asymptomatic diverticular disease according to grade

	All	No diverticular disease	Mild diverticular disease	Advanced diverticular disease	*p* value (no vs. mild)	*p* value (no vs. advanced)
Total energy intake (kilocalories) (kcal/d)	1836.5 ± 409.3	1860.2 ± 438.9	1770.4 ± 367.1	1850.0 ± 343.6	0.266	1
Meat, meat products (g/d)	121.7 ± 42.6	122.5 ± 44.4	115.2 ± 38.3	129.5 ± 41.5	0.182	0.625
Fish, crustaceans (g/d)	22.2 ± 14.7	22.5 ± 14.7	22.3 ± 17.0	21.0 ± 10.6	0.830	1
Potatoes, tubers (g/d)	56.6 ± 18.9	56.2 ± 19.3	54.5 ± 18.7	61.2 ± 17.4	0.155	0.219
Vegetables (g/d)	165.9 ± 60.3	173.2 ± 66.8	159.1 ± 49.4	147.9 ± 43.1	**0.019**	**0.020**
Fruits, nuts (g/d)	146.2 ± 73.8	149.2 ± 78.8	137.0 ± 62.2	149.0 ± 71.0	0.461	1
Milk, dairy products (g/d)	185.2 ± 103.2	192.1 ± 104.6	175.3 ± 97.2	174.1 ± 107.0	0.358	0.576
Grain, cereal products (g/d)	166.1 ± 45.6	169.8 ± 48.1	158.4 ± 44.9	163.6 ± 34.0	0.173	0.816
Protein (g/d)	69.9 ± 14.8	70.8 ± 15.8	67.4 ± 13.5	70.3 ± 12.6	0.233	1
Fat (g/d)	77.0 ± 16.4	78.0 ± 17.5	74.5 ± 14.2	77.3 ± 14.8	0.278	1
Fiber (g/d)	16.4 ± 4.4	16.8 ± 4.7	15.7 ± 3.8	16.2 ± 3.6	0.129	0.688

Bold values denote statistical significance at the *p* < 0.05 level

Variables as mean and standard deviation**.** *p* values from t-test with Bonferroni correction

In univariate analysis, subjects with any form of asymptomatic diverticular disease had significantly lower mean vegetable intake compared to subjects without diverticular disease (173.2 ± 66.8 vs. 159.1 ± 49.4 vs. 147.9 ± 43.1 g/day in participants without vs. mild vs. advanced diverticular disease respectively; all *p* < 0.05; Table 2). While participants without diverticular disease showed the highest mean fiber intake per day (16.8 ± 4.7 g/day), participants with advanced diverticular disease showed a higher fiber intake than participants with mild diverticular disease (16.2 ± 3.6 vs. 15.7 ± 3.8 g/day in participants with advanced vs. mild diverticular disease; Table 2).

While participants with advanced diverticular disease showed the highest mean intake of meat or meat products per day (129.5 ± 41.5 g/day), participants without diverticular disease showed a higher meat intake compared to participants with mild diverticular disease (122.5 ± 44.4 vs. 115.2 ± 38.3 g/day in participants without vs. mild diverticular disease; Table 2).

After adjustment for age, sex and total energy intake, a higher intake of fiber and vegetables was associated with a lower odds for any form of asymptomatic diverticular disease (OR 0.68, 95% CI [0.48, 0.95]; *p* = 0.022 and OR 0.72, 95% CI [0.53, 0.97]; *p* = 0.030 for fiber and vegetable intake respectively; Table 3), whereas high intake of meat and meat products was associated with an approximately two-fold higher odds ratio for advanced diverticular disease (OR 1.84, 95% CI [1.13, 2.99]; *p* = 0.014; Table 3). After additional adjustment for BMI, smoking behavior, alcohol consumption and physical activity only a high-fiber intake and higher intake of vegetables remained significantly associated with lower odds of asymptomatic diverticular disease (OR 0.64, 95%CI [0.44, 0.94]; *p* = 0.024 and OR 0.66, 95% CI [0.47, 0.94]; *p* = 0,023 for fiber and vegetable intake respectively Table 3). Age was significantly associated with lower meat consumption (β =  − 0.63, 95% CI [− 1.06, − 0.20], *p* = 0.004) after adjustment for total energy intake, while the presence of asymptomatic diverticular disease increased with age.

**Table 3:** Multiple regression analysis of association of dietary intake to presence of asymptomatic diverticular disease

	Model 1 (adjusted for age, sex, and total energy intake)						Model 2 (Model 1 + adjusted for BMI, alcohol consumption, smoking behavior and physical activity)					
	Outcome no versus any diverticular disease			Outcome no versus advanced diverticular disease			Outcome no versus any diverticular disease			Outcome no versus advanced diverticular disease		
	OR	95% CI	*p*	OR	95% CI	*p*	OR	95% CI	*p*	OR	95% CI	*p*
*Dietary intake*												
Meat, meat products	1.18	[0.84,1.65]	0.335	1.84	[1.13, 2.99]	**0.014**	1.07	[0.71, 1.6]	0.759	1.40	[0.77, 2.54]	0.268
Vegetables	0.72	[0.53, 0.97]	**0.030**	0.60	[0.37, 0.99]	**0.045**	0.66	[0.47, 0.94]	**0.023**	0.53	[0.29, 0.97]	**0.041**
Fruits, nuts	0.78	[0.6, 1.02]	0.072	0.74	[0.5, 1.1]	0.139	0.77	[0.58, 1.01]	0.060	0.70	[0.46, 1.06]	0.093
Fiber	0.68	[0.48, 0.95]	**0.022**	0.57	[0.34, 0.94]	**0.028**	0.64	[0.44, 0.94]	**0.024**	0.59	[0.33, 1.05]	0.074

Bold values denote statistical significance at the *p* < 0.05 level

Presented are odds ratios (OR) and 95% confidence intervals (CI) from a logistic regression model with outcome diverticular disease. Models are adjusted for age, sex, total energy intake, BMI, alcohol consumption and smoking behavior. All predictor variables were standardized (mean = 0, SD = 1) before analysis

## Discussion

We investigated the association of habitual dietary intake with the presence and extent of asymptomatic diverticular disease as characterized by MRI in a sample of a general Western population. We found that high intake of vegetables and fiber was associated with a lower prevalence of asymptomatic diverticular disease, independent of age, sex, total energy intake, BMI and lifestyle factors such as smoking behavior, alcohol consumption and physical activity. Furthermore, we found that an increased intake of meat or meat products was associated with an approximately two-fold higher odds for the prevalence of advanced diverticular disease, independent of age, sex and total energy intake, however, after additional adjustment for BMI and lifestyle factors this association attenuated. Moreover, high intake of fruits and nuts had no impact on presence or extent of asymptomatic diverticular disease. Thus, our study contributes to the evidence that there is an association between dietary intake and the presence of diverticular disease with protective effects of high-fiber diet and increased intake of vegetables.

It is still a controversy, if a low-fiber diet promotes the development of diverticular disease and its complications. So far, findings have been conflicting, as Peery et al. [16, 36] refuted the assumption of a relationship between diverticulosis and a low-fiber diet and even found that a high-fiber diet was associated with higher, rather than lower prevalence of diverticulosis, while Crowe et al. reported a protective effect of a high-fiber diet versus diverticular disease in the prospective follow-up EPIC Oxford study [19]. One possible explanation may include selection bias, as Peery et al. [36] enrolled subjects with at least one histologically verified resected adenoma prior study entry. Also, one of his studies was nested in larger studies assessing environmental and life-style factors associated with colorectal adenomas in patients undergoing an outpatient colonoscopy [16]. However, our findings are in line with Crowe et al. We investigated fiber intake in an asymptomatic cohort with no complications of diverticular disease, while Crowe et al. [19] studied the links between fiber intake and the development of complications of diverticular disease. Consequently, our results indicate that a low-fiber diet not only plays an important role in the development of complications of diverticular disease, but also in the development of diverticula.

Previously, Painter and Burkitt [2] introduced the hypothesis that a low-fiber diet was responsible for development of diverticular disease, because a low-fiber diet would cause excessive colonic pressure resulting in herniation of the mucosa through the muscle wall. In our study, we found that the presence of asymptomatic diverticular disease was significantly associated with a low-fiber diet, independent of the risk factors age, sex, total energy intake, BMI and lifestyle factors (smoking behavior, alcohol consumption and physical activity). Interestingly, we observed, that mean fiber intake was lower in participants with mild diverticular disease as compared to participants with advanced diverticular disease. Thus, one could speculate that low-fiber intake earlier in life may have contributed to the development of diverticular disease, while high-fiber intake later in life—and perhaps detected by our study—may not have a healing effect on the condition anymore. Given our detected inverse association between fiber intake and asymptomatic diverticular disease, early dietary intervention and education may be beneficial in preventing progression of the disease.

We found that an intake of vegetables was significantly inversely associated with asymptomatic diverticular disease in univariate analysis as well as after adjustment for age, sex, total energy intake, BMI and lifestyle factors (smoking behavior, alcohol consumption and physical activity).

While there have been studies, which found a lower risk of diverticular disease and its complications in vegetarian participants [19, 37], our study investigates the implications of a low vegetable intake on the presence of diverticular disease independently of a vegetarian life-style. Thus, our results may allow the assumption that a high amount of consumed vegetables may prevent the development of diverticular disease independently from other dietary habits.

Specifically, rapid bowel transit times and increased frequency of bowel movements is considered to be the possible biological mechanism through which a vegetarian and high-fiber diet lowers the risk of diverticular disease [38, 39]. A reduced transit time leads to less water being reabsorbed in the lower gastrointestinal tract resulting in larger soft stools that are easier to pass and consequently a lower intraluminal pressure and a reduced likelihood of forming diverticula [5]. The fact, that the risk of diverticular disease was found to be lower in vegetarian participants [19, 37], is in line with our findings, as an increased intake of meat or meat products was linked with an approximately two-fold increased odds of advanced diverticular disease independent of age, sex and total energy intake.

Contrary to the assumptions of previous studies, we found no association of diverticular disease with the intake of grains, fruits and nuts, confirming the findings of a prospective follow-up study of US men of Strate et al. [21], who rebuted the general presumption of a negative effect of grains, nuts and popcorn in the development of diverticular disease or diverticular complications.

Even though other lifestyle factors such as alcohol consumption, smoking and physical inactivity have been described as risk factors for diverticular disease [40, 41], we did not detect a significant association with the presence and extent of diverticular disease in univariate analysis, as previously reported [22]. However, as these factors have been described as risk factors for diverticular disease, we considered these confounders in our multiple regression analysis.

It has already been observed that age is an important risk factor for diverticular disease [3] and consistently, we previously found an association between the presence and extent of diverticular disease and higher age [22]. Notably, our findings on impact of nutrition findings persisted after adjustment for age, supporting earlier nutritional research, which has shown that diets do not change greatly over time and for many people recent diet is a reasonable reflection of diet several years or decades previously [42].

This study has potential limitations. Overall, we included 308 participants, which represents not the entire sample of the initially enrolled subjects. However, while this may have induced a potential selection bias, subjects were only excluded due to incomplete scanning protocols or insufficient image quality and no significant demographic difference was observed. The initial study design focused on the assessment of subclinical cardiovascular alterations in a Western study population, thus, the participants did not receive specific bowel preparation and the extent of diverticula in the colonic segments could have been underestimated. Data on possible symptomatic or complicated disease were not specifically collected and outcome data on the development of inflammatory alterations or complicative diverticular disease were also missing. However, no participant showed imaging indications of complicative diverticular disease. Also, the survey methodology for the assessment of habitual dietary intake only refers to a limited time period. Thus, our dietary data do not describe long-term diet and validity of general nutrition details and details about dietary habits are limited. Furthermore, longitudinal outcome data is missing, as this study represents the baseline cross-sectional study results. Hence, further large-scale studies are warranted to validate the generalizability of our findings.

## Conclusions

In conclusion, our results indicate that a high-fiber diet and an increased intake of vegetables is linked to a lower risk of asymptomatic diverticular disease, independent of age, sex, total energy intake, BMI, smoking behavior, alcohol consumption and physical activity. Furthermore, an increased intake of meat or meat products is positively associated with the occurrence of advanced diverticular disease, while an increased intake of fruits and nuts is not associated with the presence and extent of diverticular disease. Given the significant health burden of diverticular disease and its complications and the lack of reliable studies about risk factors for asymptomatic diverticular disease, our findings may contribute to a better understanding of the underlying pathomechanisms and this may have high relevance for risk stratification and the establishment of preventive measures.

## Supplementary Information

**Additional file 1.** **Table S1.** Comparison of the participants without available nutrition data to those with available nutrition data.

## Data Availability

The datasets used and/or analyzed during the current study are available from the corresponding author on reasonable request.

## Abbreviations

BMI: Body mass index
CI: Confidence interval
FFQ: Food frequency questionnaire
KORA: Cooperative Health Research in the Augsburg Region, Germany
MRI: Magnetic resonance imaging
OR: Odds ratio
