# Supplementary material for: Dietary habits and the presence and degree of asymptomatic diverticular disease by magnetic resonance imaging in a Western population: a population-based cohort study
Source: Nutr Metab (Lond). 2021 Jul 16;18:73. doi: 10.1186/s12986-021-00599-4 (PMC8283990; doi:10.1186/s12986-021-00599-4)
Supplement: Supplementary file 1 — Additional file 1. Table S1. Comparison of the participants without available nutrition data to those with available nutrition data. [file 12986_2021_599_MOESM1_ESM.docx]

**Additional material**

**Additional file 1.** Comparison of the participants without available nutrition data to those with available nutrition data.

|  | Participants without nutrition data | Participants with nutrition data | p-value |
| --- | --- | --- | --- |
|  | N = 85 | N = 308 |  |
| Age (years) | 56.3 ± 9.6 | 56.4 ± 9.1 | 0.934 |
| Men | 54 (63.5%) | 172 (55.8%) | 0.252 |
| BMI (kg/m^2^) | 28.7 ± 4.7 | 28.0 ± 5.0 | 0.251 |
| normal | 16 (18.8%) | 89 (28.9%) |  |
| overweight | 41 (48.2%) | 127 (41.2%) | 0.174 |
| obese | 28 (32.9%) | 92 (29.9%) |  |
| Alcohol, g/d | 19.1 ± 24.2 | 18.2 ± 23.9 | 0.773 |
| Physical activity |  |  |  |
| inactive | 37 (43.5%) | 122 (39.6%) | 0.598 |
| active | 48 (56.5%) | 186 (60.4%) |  |
| Smoking |  |  |  |
| never-smoker | 30 (35.3%) | 114 (37.0%) | 0.806 |
| ex-smoker | 36 (42.4%) | 135 (43.8%) |  |
| smoker | 19 (22.4%) | 59 (19.2%) |  |
| Total Cholesterol (mg/dl) | 218.7 ± 37.3 | 217.4 ± 36.3 | 0.778 |
| HDL Cholesterol (mg/dl) | 59.6 ± 17.2 | 62.5 ± 17.8 | 0.181 |
| LDL Cholesterol (mg(dl) | 140.3 ± 31.1 | 139.2 ± 33.5 | 0.793 |
| Triglycerides (mg/dl) | 145.7 ± 102.7 | 128.2 ± 79.7 | 0.095 |
| Vitamin D (ng/ml) | 25.3 ± 13.0 | 23.1 ± 11.4 | 0.135 |
| Systolic blood pressure (mmHg) | 123.1 ± 18.0 | 120.0 ± 16.4 | 0.137 |
| Diastolic blood pressure (mmHg) | 77.0 ± 10.2 | 74.8 ± 10.0 | 0.079 |
| Antihypertensive Medication | 17 (20.0%) | 84 (27.3%) | 0.223 |
| Antithrombotic drugs | 3 (3.5%) | 20 (6.5%) | 0.435 |
| Degree of diverticular disease | | | |
| No | 44 (51.8%) | 185 (60.1%) | 0.074 |
| Mild | 19 (22.4%) | 76 (24.7%) |  |
| Advanced | 22 (25.9%) | 47 (15.3%) |  |

Variables as mean and standard deviation or number and percentage. P-values from t-test or χ^2^-test, where appropriate.
